# Osimertinib or EGFR-TKIs/chemotherapy in patients with EGFR-mutated advanced nonsmall cell lung cancer: A meta-analysis

**DOI:** 10.1097/MD.0000000000017705

**Published:** 2019-10-25

**Authors:** Lei Huang, Hao Huang, Xiao-Ping Zhou, Jin-Feng Liu, Chun-Rong Li, Min Fang, Jun-Rong Wu

**Affiliations:** a Department of Clinical Laboratory, The Affiliated Tumor Hospital of Guangxi Medical University; b Department of Clinical Laboratory, The First Affiliated Hospital of Guangxi University of Chinese Medicine, Nanning, Guangxi, China.

**Keywords:** chemotherapy, epidermal growth factor receptor, mutation, nonsmall cell lung cancer, Osimertinib

## Abstract

**Background:**

The aim of this meta-analysis is to investigate the impact of Osimertinib on treatment efficacy in advanced nonsmall cell lung cancer (NSCLC).

**Methods:**

Trials comparing Osimertinib against epidermal growth factor receptor tyrosine kinase inhibitors (EGFR-TKIs)/chemotherapy in patients with NSCLC with an epidermal growth factor receptor (EGFR) mutation were included, and the pooled data for progression-free survival (PFS), overall survival (OS), overall response rate (ORR), disease control rate (DCR), and adverse events (AEs) were analyzed.

**Results:**

Analysis results based on 6 eligible trials showed that Osimertinib significantly improved the overall PFS (hazard ratio [HR] = 0.38, 95% confidence interval [CI] = 0.29–0.50), improved the OS (HR = 0.66, 95% CI = 0.48–0.89), increased the ORR (odds ratio [OR] = 1.76, 95% CI = 1.14–2.72), increased the overall DCR (OR = 1.18, 95% CI = 1.02–1.37), and reduced the grade 3 or greater AEs (relative ratio [RR] = 0.50, 95% CI = 0.33–0.75) in all subgroups except in the ORR in the Exon 19 deletion (Ex19del) and/or L858R subgroup. Compared to patients with Ex19del and/or L858R mutation, patients with the T790M mutation had the benefits of a greater PFS (41.7%), a greater ORR (80.0%), a greater DCR (71.2%), and fewer grade 3 or greater AEs (70.7%) (each *P* < .05). Race, sex, age, EGFR mutation, and smoking history may significantly predict additional benefits from Osimertinib, but there were no significant differences between subgroups stratified by these clinical characteristics.

**Conclusions:**

Osimertinib showed greater treatment benefit for patients with NSCLC with EGFR mutation than EGFR-TKIs/chemotherapy, especially for T790M mutation-positive patients.

## 1 Introduction

Lung cancer is still one of the most commonly diagnosed cancers and a major cause of cancer-related mortality in men and women.^[1]^ In addition, lung cancer places enormous pressures on the quality of living and economic capability of patients. Most lung cancers are nonsmall cell lung cancer (NSCLC), and metastatic disease occurs in more than half of patients. With the poor prognosis of advanced NSCLC, the issue of treatment for advanced NSCLC has received considerable critical attention.^[2]^ Epidermal growth factor receptor tyrosine kinase inhibitors (EGFR-TKIs) are considered the standard first-line therapies for NSCLC patients with EGFR mutations.^[3,4]^ Previous studies evaluating treatment effects in advanced NSCLC patients with EGFR mutations have shown that EGFR-TKIs are more effective than standard platinum-based chemotherapy with regard to progression-free survival (PFS), overall response rate (ORR), characteristics, and quality of life.^[5–7]^ Patients with positive mutations of the EGFR gene can obtain a median remission time of approximately 10 months from first-generation EGFR-TKIs, but the problem of acquired drug resistance remains to be solved. The most important molecular mechanism is the T790M secondary mutation in exon 20 of the EGFR gene. The second-generation of EGFR-TKIs further validates the advantages of EGFR-TKIs over standardized therapy and refines the differences in efficacy among different types of EGFR gene mutations. The efficacy of second-generation EGFR-TKIs seems to be better than that of first-generation EGFR-TKIs, but this efficacy is not satisfactory in overcoming acquired drug resistance; in addition, the toxic side effects of second-generation EGFR-TKIs are more worrying than those of first-generation EGFR-TKIs.^[8,9]^ KRAS is a signaling pathway downstream of EGFR, and its mutation is one of the mechanisms of tumor formation such as lung cancer. Data from several studies suggested that EGFR and KRAS mutations were associated with poor prognosis of NSCLC patients.^[10,11]^ EGFR-TKs show greater treatment benefits for NSCLC patients with EGFR mutation rather than KRAS mutation. EGFR and KRAS mutations are mutually exclusive, and the detection of KRAS mutation is useful to predict the effect of EGFR-TKI treatment in patients with EGFR mutation. It has been reported that the efficacy of new EGFR-TKs for patients with EGFR mutation can be evaluated by OSAR model.^[12,13]^

Osimertinib (Tagrisso, AstraZeneca) is a highly selective third-generation EGFR-TKI that is an effective target drug against acquired T790M resistance from EGFR-TKIs and has the lowest activity against wild-type EGFR.^[14,15]^ Results from phase II trials and case reports have established that Osimertinib had a higher ORR and longer PFS in patients with advanced NSCLC who were T790M mutation positive than in T790M mutation-negative advanced NSCLC patients.^[16,17]^ Recently, considerable literature has focused on the theme of clinical efficacy and safety when comparing Osimertinib against EGFR-TKIs or chemotherapy in this genetically distinct subset of NSCLC.^[18,19]^ Despite statistically significant PFS benefit, few researchers have been in a position to draw on any systematic conclusions on overall survival (OS), and previously published studies on the safety and adverse events (AEs) of Osimertinib are not consistent.

In addition, the effect of different types of EGFR mutations on the efficacy of Osimertinib is also of concern. Several randomized controlled trials (RCTs) that focused on Osimertinib used for advanced NSCLC patients have been released; however, the clinical benefits of Osimertinib in advanced NSCLC patients with different EGFR mutation types as well as other clinical characteristics remain unresolved. For treatment differences between subgroups of patients with different EGFR mutation types, discrete trials have not been designed or have not provided sufficient motivation. Therefore, to solve these problems, a meta-analysis is needed.

In this study, the main objective was to determine the effect of Osimertinib against EGFR-TKIs/chemotherapy on PFS. The secondary objectives were to evaluate OS, ORR, disease control rate (DCR), and AEs between Osimertinib and EGFR-TKIs or chemotherapy. This work will generate fresh insights into the treatment effects of Osimertinib in patients with advanced NSCLC with EGFR mutations.

## 2 Patients and methods

### 2.1 Search strategy

The included studies in this thesis were trials that compared Osimertinib against EGFR-TKIs/chemotherapy in advanced NSCLC patients with EGFR mutations. A comprehensive search was conducted on PubMed, Embase, the Cochrane Central Register of Controlled Trials, and Web of Science databases using the following search terms: non-small-cell lung cancer, NSCLC, lung cancer, lung neoplasms, Epidermal Growth Factor Receptor, EGFR, EGFR-TKIs, Afatinib, Gefitinib, Dacomitinib, Erlotinib, AZD-9291, mesylate, Tagrisso, Osimertinib, meta-analysis, systematic review, and clinical trials. In addition, unpublished studies were identified and retrieved from the American Society of Clinical Oncology, the European Society of Medical Oncology, and the World Conference on Lung Cancer. The period of retrieval was from January 1, 2015 to December 31, 2018. Two investigators independently performed the searches.

### 2.2 Eligibility criteria

Studies that met the following criteria were included for analysis in the meta-analysis: patients were diagnosed with NSCLC with EGFR mutations at baseline; trials compared Osimertinib against EGFR-TKIs/chemotherapy; studies were RCTs, cohort, or case–control study design; studies reported PFS or provided data for the calculation of hazard ratios (HRs) with their 95% confidence intervals (CIs); and studies reported data on safety and AEs. Letters, review articles, and case reports were not included in the present study. Two independent researchers reviewed the abstracts separately and selected articles for complete manuscript review based on the inclusion criteria. The quality of the RCTs was evaluated using the Cochrane Handbook for Systematic Reviews of Interventions (version 5.1.0).^[20]^ Discrepancies between the reviewing authors were reconciled by consensus.

### 2.3 Data extraction

The following data from each eligible study were abstracted: baseline characteristics as follows: first author, publication year, number of participants, EGFR mutation (T790M, Exon 19 deletion [Ex19del] and/or L858R) subtypes, study design, type of treatment, median age, proportion of female participants, and proportion of participants that were never smokers; survival outcomes as follows: median OS, median PFS, median duration of PFS, and HRs with 95% CIs for OS or PFS; response to treatment, including ORR, DCR; and AE data, including any grade AE and grade 3 or greater AEs. Two authors (Lei Huang and Xiao-Ping Zhou) extracted relevant data independently, and a third author (Min Fang) was consulted to resolve discrepancies when necessary.

### 2.4 Statistical analysis

The principle aim of this study was to investigate the PFS when comparing Osimertinib against EGFR-TKIs/chemotherapy in patients with advanced NSCLC with EGFR mutations. The secondary objectives of the study were to determine the OS, ORR, DCR, and AEs between the 2 treatment regimens.

The summary of the effects of the PFS and OS were calculated as the HR and 95% CI, and an HR < 1 reflected Osimertinib in favor of longer OS or PFS. The effects of the ORR and DCR were measured as the odds ratio (OR) and 95% CI, and an OR > 1 reflected Osimertinib therapy in favor of higher ORR or DCR. The analysis of the impacts of the AEs was assessed as the relative ratio (RR) and 95% CI, and an RR < 1 reflected Osimertinib therapy in favor of lower AEs. A chi-squared test was used to evaluate the heterogeneity in the study, and moderate and significant statistical heterogeneity were defined as 50% < *I*^2^ ≤ 75% and *I*^2^ > 75%, respectively. The fixed effects method was first conducted on pooled data, and the random effects method was performed when significant heterogeneity was found across studies. The source of the heterogeneity was explored by subgroup analysis based on EGFR mutation status (treatment regimen). Publication bias was performed via Begg and Egger tests.^[21,22]^ All statistical analyses were performed with the STAT 14.0 package (Stata Corporation, College Station, TX). Statistically significant is defined as *P* < .05 with 2-tailed analysis.

All analyses were based on previous published studies; thus, no ethical approval and patient consent are required.

## 3 Results

### 3.1 Clinicopathologic characteristics of the patients

**Figure 1:**
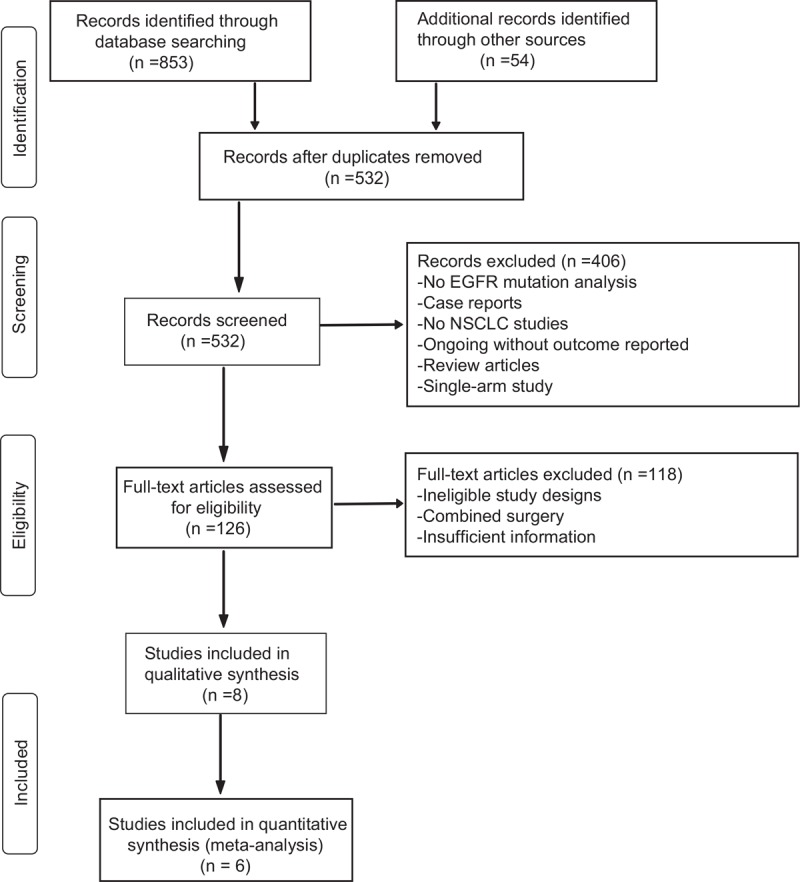
Flowchart of study selection.

A total of 6 eligible RCTs^[18,19,23–26]^ that provided data from 1433 patients with advanced, EGFR-mutated NSCLC were included in this meta-analysis. The retrieval process of the present study is presented in Figure 1. Three trials recruited 804 patients with Ex19del and/or L858R mutation, and 3 other trials recruited 629 patients with other common EGFR mutations, including T790M. Each RCT was an open label, phase 3 trial, and had been released. The risk of bias assessed with the Cochrane Handbook for Systematic Reviews of Interventions was low, although 3 included trials reported more individual patient data than previously published data from the AURA3 and FLAURA trials. Three RCTs recruited patients who were not previously treated, and 3 studies recruited patients who had disease progression after first-line anticancer therapy; these anticancer drugs were mainly Afatinib, Gefitinib, and Erlotinib. For the type of treatment, one trial compared Osimertinib against docetaxel–bevacizumab. Two trials compared Osimertinib against platinum-pemetrexed, and 3 trials compared Osimertinib against gefitinib and/or erlotinib.

The participants had median ages of 48.6 to 69.0 years and were from both Osimertinib and EGFR-TKIs/chemotherapy backgrounds; most patients were never smokers (range 52.7%–82.4%). The rates of female participants ranged from 50.9% to 71.6%. Table 1 summarizes the clinical characteristics of eligible participants. As shown in Table 1, patients in the T790M mutation subgroup were treated with Osimertinib or chemotherapy (platinum-pemetrexed, docetaxel, and/or bevacizumab), and those in the Ex19del and/or L858R mutation subgroup were treated with Osimertinib or EGFR-TKIs (gefitinib and/or erlotinib); therefore, the pooled results of the subgroup based on treatment regimens were the same as those based on the EGFR mutation status.

**Table 1:**
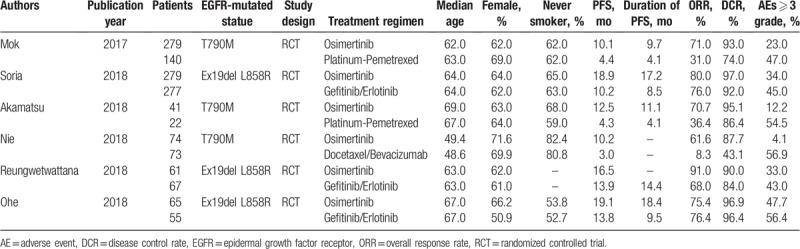
Characteristics of patients in constituent trials.

### 3.2 Pooled analysis of PFS

**Figure 2:**
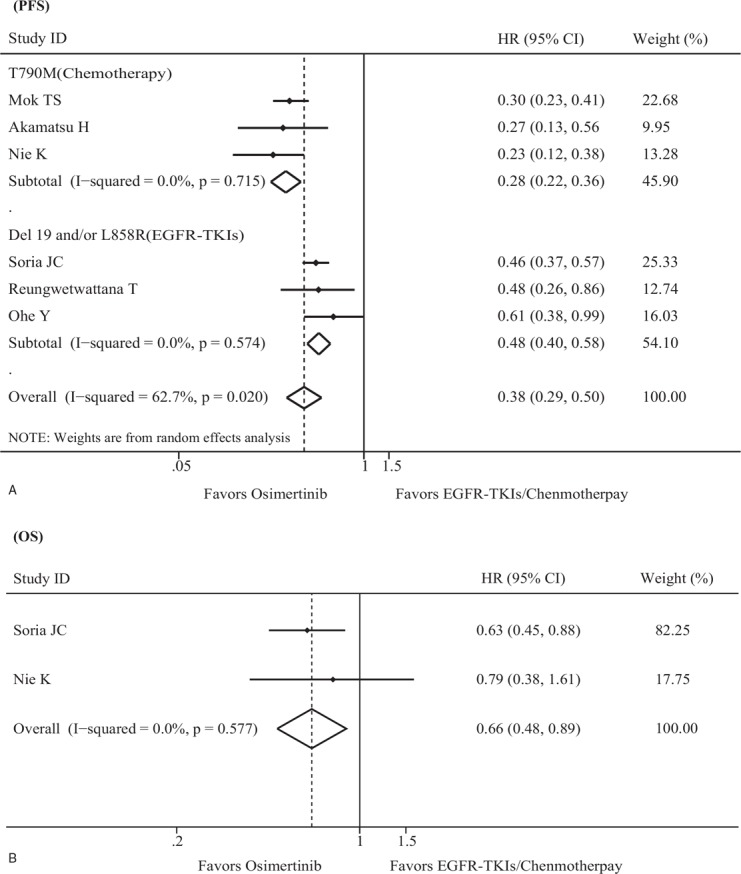
Randomized treatment effect on PFS and OS. (A) Forest plot of the effect of treatment on PFS. (B) Forest plot of the effect of treatment on OS. CI = confidence interval, EGFR-TKI = epidermal growth factor receptor tyrosine kinase inhibitor, HR = hazard ratio, OS = overall survival, PFS = progression-free survival.

PFS data were accessible for all included trials, with data for a total of 1433 patients. Significant heterogeneity was found across all eligible studies (*I*^2^ = 62.7%, *P* = .020). Therefore, the HR and 95% CI for PFS were pooled using a random effects model. As shown in Figure 2A, Osimertinib was associated with significantly longer PFS than EGFR-TKIs/chemotherapy (HR, 0.38; 95% CI, 0.29–0.50; *P* < .001) in the full analysis set. In the T790M mutation subgroup, the HR for PFS was 0.28 (95% CI, 0.22–0.36; *P* < .001), and no heterogeneity was observed. In the Ex19del and/or L858R mutation subgroup, the HR for PFS was 0.48 (95% CI, 0.40–0.58; *P* < .001), and no heterogeneity was observed. Compared with EGFR-TKIs/chemotherapy, Osimertinib demonstrated a 41.7% greater benefit for the T790M mutation subgroup than the Ex19del and/or L858R mutation subgroup (*P* = .001). Sensitivity analyses showed that no trial significantly affected the outcome. The Begg and Egger tests showed no evidence of publication bias (each *P* < .05).

**Figure 3:**
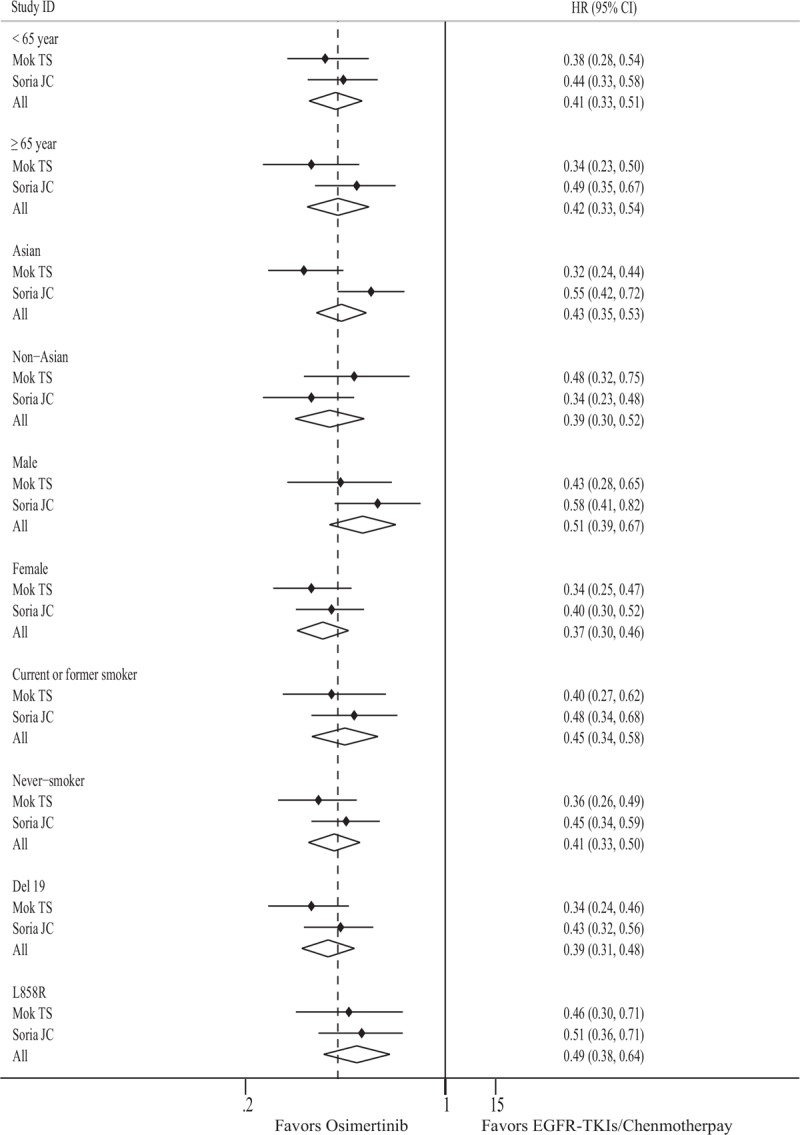
Forest plot of the effect of treatment on PFS in subgroups of patients according to clinical characteristics. CI = confidence interval, EGFR-TKI = epidermal growth factor receptor tyrosine kinase inhibitor, HR = hazard ratio, PFS = progression-free survival.

In the present study, 2 eligible trials performed subgroup analysis of PFS based on clinical characteristics.^[18,19]^ A summary of statistics is presented in Figure 3 and shows that age, race, sex, smoking history, and EGFR mutation types may significantly predict additional benefit from Osimertinib; however, there were no significant differences between subgroups stratified by race (Asian vs non-Asian, *P* = .590), sex (male vs female, *P* = .063), age (<65 years vs ≥65 years, *P* = .908), EGFR mutation (Ex19del vs L858R, *P* = .184), and smoking history (never smoker vs current or former smoker, *P* = .618).

### 3.3 Pooled analysis of OS

OS data were only available for 2 trials^[19,24]^ with 703 patients included as the remaining 4 studies did not report the data for OS. The pooled results from the fixed effects model demonstrated that patients who received Osimertinib had a longer OS than those who received EGFR-TKIs/chemotherapy (HR = 0.66; 95% CI = 0.48–0.89; *P* = .007) (Fig. 2B) without heterogeneity (*I*^2^ = 0.0%, *P* = .557). Sensitivity analyses and publication bias were not conducted as only 2 trials included the data analysis for OS.

### 3.4 Pooled analysis of ORR

**Figure 4:**
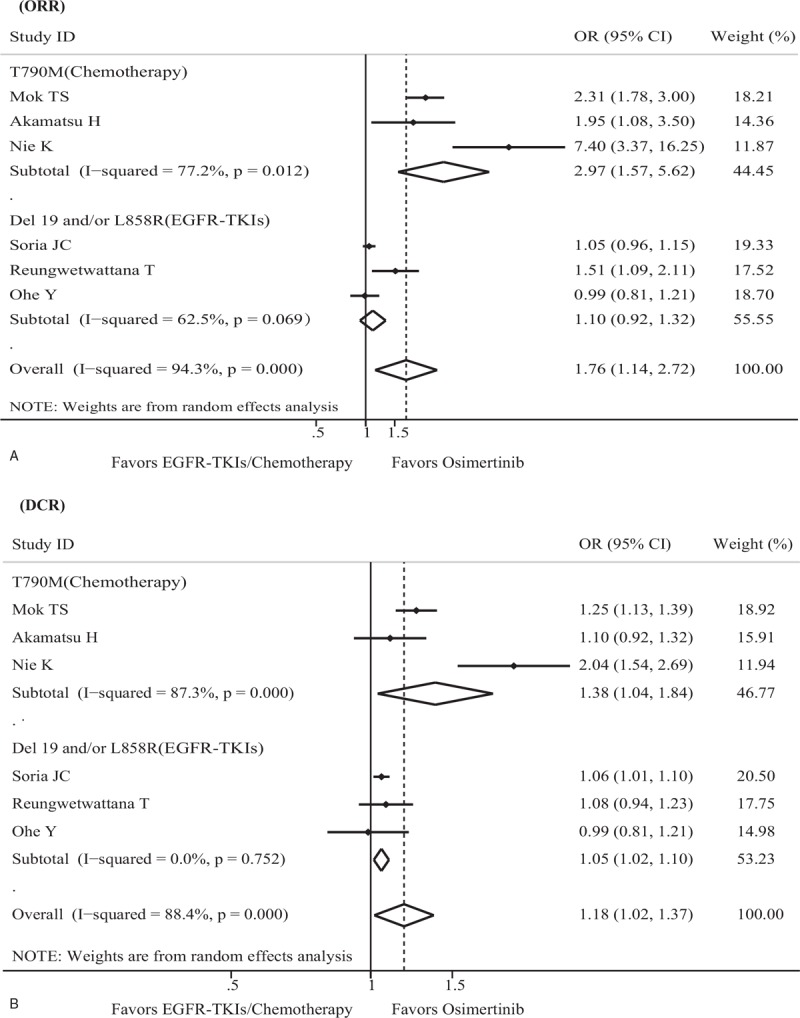
Randomized treatment effect on ORR and DCR. (A) Forest plot of the effect of treatment on the ORR. (B) Forest plot of the effect of treatment on the DCR. CI = confidence interval, DCR = disease control rate, EGFR-TKI = epidermal growth factor receptor tyrosine kinase inhibitor, OR = odds ratio, ORR = overall response rate.

The tumor ORRs were obtained from all eligible studies that included 1431 participants. High heterogeneity across studies was detected using the fixed effects model; therefore, the random effects model was used to pool the ORR data (*I*^2^ = 94.3%, *P* < .001). The ORR of patients treated with Osimertinib was 73.2% (584/798), whereas the ORR of those treated with EGFR-TKIs/chemotherapy regimens was 53.6% (339/633); further analysis indicated that Osimertinib therapy could improve ORR for advanced NSCLC patients with EGFR mutations compared with EGFR-TKIs/chemotherapy (OR = 1.76; 95% CI = 1.14–2.72; *P* = .011) (Fig. 4A). High heterogeneity was also found in both the T790M mutation subgroup (*I*^2^ = 77.2%, *P* = .012) and the Ex19del and/or L858R mutation subgroup (*I*^2^ = 62.9%, *P* = .069). Pooled results from the subgroup analysis based on the EGFR mutation types (T790M mutation subgroup and Ex19del and/or L858R mutation subgroup) demonstrated that significant positive effects were also observed in the T790M mutation subgroup (OR = 2.97; 95% CI = 1.57–5.62; *P* = .001) rather than in the Ex19del and/or L858R mutation subgroup (OR = 1.10; 95% CI = 0.92–1.32; *P* = .279). Compared with EGFR-TKIs/chemotherapy, Osimertinib demonstrated 80.0% greater benefit for the T790M mutation subgroup than the Ex19del and/or L858R mutation subgroup (*P* = .001). Sensitivity analysis indicated that the trial of Nie et al^[24]^ was the primary source of heterogeneity; moreover, our findings were statistically robust after this trial was excluded. No publication bias was detected using either Begg or Egger test (each *P* < .05).

### 3.5 Pooled analysis of DCR

The tumor DCR was drawn from all included studies with 1431 patients. High heterogeneity across studies was observed in both the full analysis set (*I*^2^ = 88.4%, *P* < .001) and the T790M mutation subgroup (*I*^2^ = 87.3%, *P* < .001), so the random effects model was suitable for pooling the OR of DCR. The DCR of patients treated with Osimertinib was 92.5% (738/798), whereas the DCR of those treated with EGFR-TKIs/chemotherapy was 80.1% (507/633), which indicated that the Osimertinib therapy subgroup could obtain higher a DCR than the EGFR-TKIs/chemotherapy subgroup in advanced NSCLC patients with EGFR mutations, regardless of the full analysis set or subgroup set (full analysis: OR = 1.18, 95% CI = 1.02–1.37, *P* = .028; T790M mutation subgroup: OR = 1.38, 95% CI = 1.04–1.84, *P* = .028; Ex19del and/or L858R mutation subgroup: OR = 1.05, 95% CI = 1.02–1.10, *P* = .006) (Fig. 4B). Compared with EGFR-TKIs/chemotherapy, Osimertinib demonstrated 71.2% greater benefit for the T790M mutation subgroup than the Ex19del and/or L858R mutation subgroup (*P* = .005). Sensitivity analyses showed that no trial dramatically affected our findings. No evidence of publication bias was displayed with the Begg and Egger tests (each *P* < .05).

### 3.6 Pooled analysis of AEs

**Figure 5:**
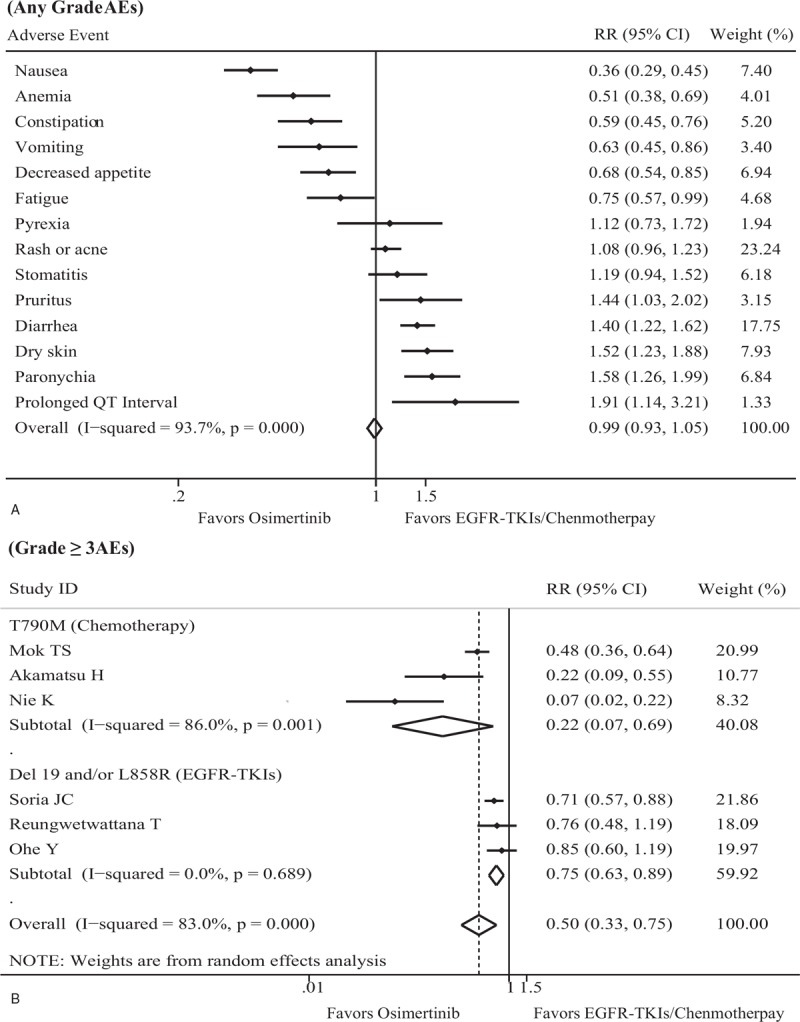
Pooled analysis and subgroup analysis of AEs. (A) Forest plot of the safety of treatment on any AE. (B) Forest plot of the safety of treatment on grade 3 or greater AEs. AE = adverse event, CI = confidence interval, EGFR-TKI = epidermal growth factor receptor tyrosine kinase inhibitor, RR = relative ratio.

Toxicity profile analyses for eligible trials are presented in Figure 5. For any grade of AEs, the incidence of nausea, anemia, constipation, vomiting, decreased appetite, and fatigue in the Osimertinib therapy subgroup was significantly lower than in the EGFR-TKIs/chemotherapy subgroup. However, the Osimertinib therapy subgroup exhibited a higher proportion of pruritus, diarrhea, dry skin, paronychia, and prolonged QT interval than the EGFR-TKIs/chemotherapy subgroup. No significant differences between the 2 subgroups for the rates of pyrexia, rash or acne, and stomatitis were displayed (Fig. 5A).

All eligible trials documented the proportion of grade 3 or greater AEs. There was significant heterogeneity in both the full trial set (*I*^2^ = 83.0%, *P* < .001) and the T790M mutation subgroup (*I*^2^ = 86.0%, *P* = .001), while no heterogeneity was found in the Ex19del and/or L858R mutation subgroup (*I*^2^ = 0.0%, *P* = .689). The incidence of grade ≥3 AEs in the Osimertinib subgroup was 26.4%, and the proportion of grade ≥3 AEs in the EGFR-TKIs/chemotherapy subgroup was 48.1%. Pooled data revealed that patients treated with Osimertinib were less likely to have grade 3 or greater AEs than those treated with EGFR-TKIs//chemotherapy, regardless of the full analysis set or subgroup set (full analysis: RR = 0.50, 95% CI = 0.33–0.75, *P* = .001; T790M mutation subgroup: RR = 0.22, 95% CI = 0.07–0.69, *P* = .010; Ex19del and/or L858R mutation subgroup: RR = 0.75, 95% CI = 0.63–0.89, *P* = .001) (Fig. 5B). Compared with the EGFR-TKIs/chemotherapy subgroup, the Osimertinib therapy subgroup demonstrated a 70.7% lower incidence of grade 3 or greater AEs in patients with the T790M mutation than in patients with the Ex19del and/or L858R mutation (*P* = .040). No significant heterogeneity was observed from the sensitivity analysis, and publication bias was not found using either the Begg or Egger test (each *P* < .05).

## 4 Discussion

In the present study, compared with EGFR-TKIs/chemotherapy, Osimertinib treatment reduced the risk of disease progression or death by 62% and reduced the risk of over survival by 34%. In the total analysis of response and AEs, Osimertinib contributed to a 63% greater ORR, a 70% greater DCR, and a 50% reduction in grade 3 or greater AEs than the EGFR-TKIs/chemotherapy regimen. Other crucial findings showed that Osimertinib demonstrated better efficacy and safety for advanced NSCLC patients with the T790M mutation than for those with the Ex19del and/or L858R mutation.

In reviewing the literature, NSCLC patients receiving EGFR-TKI therapy experienced longer PFS than those treated with chemotherapy,^[7,27]^ and one interesting finding in these 2 articles was that EGFR-TKIs demonstrated greater benefit with the Ex19del and/or L858R mutation than chemotherapy. Using data from 6 RCTs, this meta-analysis found that participants who received Osimertinib treatment had longer PFS than those treated with EGFR-TKIs/chemotherapy; one notable finding that emerged from the analysis was that treatment with Osimertinib improved the prognosis more in patients with the T790M mutation than in patients with the Ex19del and/or L858R mutation. This finding, while preliminary, suggests that Osimertinib may be considered the drug of choice for patients with T790M-positive tumors. However, the observed difference between the Ex19del and L858R mutations in this study was not explored, and a possible explanation for these results may be the lack of adequate related articles and information included in this study.

Following disease progression, the findings were unexpected and suggested that patients randomly assigned to the Osimertinib therapy subgroup had significantly longer OS than those assigned to the EGFR-TKIs/chemotherapy subgroup. Although only 2 trials reported related data for analysis, one of the most common reasons for this is that the use of Osimertinib as a treatment for NSCLC has started only in recent years, and a number of studies are ongoing; thus, a lack of sufficient data for OS was found. Subgroup analysis for OS was not performed as not enough related information was reported in the included studies for the present study. This finding will undoubtedly be scrutinized, but there are some immediate dependable conclusions for the benefit of Osimertinib in the treatment of NSCLC. In accordance with the present results, a previous study demonstrated that the median OS of NSCLC patients who received Osimertinib was 26.8 months (95% CI, 24.0–29.1 months),^[28]^ which seems to be longer than EGFR-TKIs and chemotherapy reported in a previous meta-analysis^[7]^; however, the observed difference in OS between EGFR-TKIs and chemotherapy in the 2 previous meta-analyses was not significant.^[7,27]^ Further studies that determine the relative OS with Osimertinib versus EGFR-TKIs/chemotherapy in EGFR-mutated NSCLC will need to be performed.

Experimental evidence for the response to treatment was also determined in this meta-analysis. Interestingly, there were also differences in the ratios of OR and DC between Osimertinib and EGFR-TKIs/chemotherapy in EGFR-mutated NSCLC. The ORR and DCR in this study were very positive for Osimertinib than for EGFR-TKIs/chemotherapy. Another important finding was that Osimertinib demonstrated greater benefit for the T790M mutation subgroup than the Ex19del and/or L858R mutation subgroup for both ORR and DCR. In addition, the ORR for Osimertinib in the current study was 78.2%, which was significantly higher than that of EGFR-TKIs/chemotherapy. This difference was also noted when the Nie trial^[24]^ was included in this study. The higher ORR of Osimertinib in EGFR-mutated advanced NSCLC also agreed with previous observations, which showed that the ORR for participants receiving Osimertinib as a first-line treatment was 67% in the 80-mg group, 87% in the 160-mg group, and 77% across the 2 doses.^[29]^

Interestingly, as shown in Table 1, the median duration of PFS seemed to be longer in participants treated with Osimertinib (range 9.4–18.4 months) than in those receiving EGFR-TKIs/chemotherapy (range 4.1–14.4 months). It is unfortunate that the statistical analysis of the median duration of PFS was not reached for the only 2 trials that reported the duration of PFS and 95% CI. A longer median duration of PFS means that patients underwent more long-term treatment, had a greater number of treatment cycles, experienced longer PFS and OS, and had higher ORR and DCR. As mentioned in the literature review, the survival rates of advanced NSCLC patients with the T790M mutation were treated with Osimertinib were 80% at 12 months, 55% at 24 months, and 37% at 36 months.^[28]^ These results indicate that Osimertinib may significantly improve the survival in advanced NSCLC patients with EGFR mutation-positive tumors.

With regard to the safety analysis, eligible trials reported that toxicities were manageable, tolerable, and predictable. One unanticipated finding was that Osimertinib was associated with a somewhat lower rate of grade 3 or greater AEs than EGFR-TKIs/chemotherapy. The data in Figure 5B shows that there is a clear trend of decreasing grade 3 or greater AEs in NSCLC patients with the T790M mutation. However, 2 trials^[25,26]^ included in the present study indicated that no significant differences for grade 3 or greater AEs were found between Osimertinib and EGFR-TKIs in patients with the Ex19del and/or L858R mutation. As mentioned in the literature review, the conclusion on grade 3 or greater AEs for EGFR-TKIs against chemotherapy in EGFR mutation-positive NSCLC patients is inconsistent.^[6,16]^ These results provide further support for the hypothesis that NSCLC patents with the T790M mutation receiving Osimertinib could have fewer grade 3 or greater AEs than those receiving EGFR-TKIs/chemotherapy.

There are several strengths in this meta-analysis. Six RCTs were reviewed and analyzed comprehensively, and PFS data were used to investigate the research problems. As Osimertinib is an irreversible EGFR-TKI with higher activity against the T790M mutation-positive tumors than other NSCLC mutations, a subgroup analysis was conducted based on the EGFR mutation status (T790M vs Ex19del and/or L858R); the findings for Osimertinib therapy were compared with those for EGFR-TKIs/chemotherapy and showed that T790M mutation-positive patients treated with Osimertinib had a longer PFS, a higher ORR, a higher DCR, and a lower incidence of grade 3 or greater AEs than patients with the Ex19del and/or L858R mutation. In addition, the present study comprehensively evaluated survival outcomes (including PFS and OS), response to treatment (containing ORR and DCR), and AEs between Osimertinib and EGFR-TKIs/chemotherapy in advanced NSCLC patients with EGFR mutations.

There are also limitations to this study. The present study has fewer eligible trials. A possible explanation for this might be that Osimertinib could be considered a new drug of choice for EGFR mutation-positive advanced NSCLC, and some investigations are ongoing. The observed significant heterogeneity in the sensitivity analysis could be attributed to the Nie trial,^[24]^ although the exclusion of this trial did not dramatically change our findings, and these findings should be interpreted with caution. The Ex19del and L858R mutations have been amalgamated into a single subgroup when the subgroup analysis was conducted for EGFR mutation status. There may be inconsistency in the efficacy and safety between patients with the Ex19del mutation and those with the L858R mutation; however, the most notable finding to emerge from the analysis was that T790M mutation-positive tumors prompted our interest. The association between the EGFR mutation status and baseline clinical characteristics, such as age, sex, histology, or smoking status, has not been investigated.

When these results are interpreted, some caution should be taken. There was significant heterogeneity in the T790M mutation subgroup when analyzing ORR, DCR, and grade 3 AEs between Osimertinib and EGFR-TKIs/chemotherapy, and further studies with more focus on Osimertinib treatment in advanced NSCLC patients with T790M mutation-positive tumors are therefore suggested. Subgroup analysis of PFS based on clinical characteristics was derived from only 2 eligible trials.

Several important clinical and research implications were suggested from our findings. An implication of these findings is the possibility that advanced NSCLC patients with T790M mutation-positive tumors obtained more clinical benefits from Osimertinib than those with tumors with the Ex19del and/or L858R mutation, suggesting that the EGFR mutation status of patients should be determined before starting treatment. This finding has important implications for further research on the effects of Osimertinib, particularly for tumors with the T790M mutation.

Another potential use of these findings is that Osimertinib could be considered a first-line treatment of EGFR mutation-positive advanced NSCLC tumors, particularly advanced NSCLC patients with T790M mutation-positive tumors, as the EGFR T790M mutation is the most common genetic change after resistance to first-line EGFR-TKI therapy.

Despite its limitations, the results of this investigation show that Osimertinib significantly prolonged PFS and OS, raised ORR and DCR, and decreased the incidence of grade 3 or greater AEs in advanced NSCLC patients with common EGFR mutations compared with EGFR-TKIs/chemotherapy. Osimertinib overcomes T790M-acquired drug resistance and has excellent efficacy and safety, which could be considered as the optimization drug for advanced NSCLC patients with T790M mutation-positive tumors. Larger studies and further clinical trials in this field are warranted to confirm our findings. In addition, the optimal timing, drug sequence and new drug resistance mutations, such as the C797S and KRAS mutations, still need to be further explored.

## Acknowledgments

The authors thank Professor Bo Zhu, Dr Yi-Feng Tao, and Dr Zhong-Qing Chen (Department of Clinical Laboratory, the Affiliated Tumor Hospital of Guangxi Medical University) for the integrity and accuracy of the data analysis.

## Author contributions

**Conceptualization:** Jun-Rong Wu.

**Data curation:** Lei Huang, Hao Huang, Xiao-Ping Zhou.

**Formal analysis:** Xiao-Ping Zhou, Jin-Feng Liu.

**Funding acquisition:** Min Fang.

**Investigation:** Lei Huang, Hao Huang.

**Methodology:** Xiao-Ping Zhou, Jin-Feng Liu.

**Project administration:** Min Fang.

**Resources:** Chun-Rong Li.

**Software:** Min Fang.

**Supervision:** Jun-Rong Wu.

**Validation:** Jin-Feng Liu.

**Visualization:** Chun-Rong Li.

**Writing – review & editing:** Jun-Rong Wu.

**Writing – original draft:** Lei Huang.
